# Divergent interpersonal neural synchronization patterns in the first, second language and interlingual communication

**DOI:** 10.1038/s41598-023-35923-w

**Published:** 2023-05-29

**Authors:** Yanqin Feng, Yuan Liang, Yi Zhang, Xu Duan, Jie Zhang, Hao Yan

**Affiliations:** 1 School of Foreign Languages, Xidian University, Xi’an, China; 2 Key Laboratory for Artificial Intelligence and Cognitive Neuroscience of Language, Xi’an International Studies University, Xi’an, China; 3 Department of Radiation Medicine, Air Force Military Medical University, Xi’an, China

**Keywords:** Neuroscience, Psychology

## Abstract

An accumulating number of studies have highlighted the importance of interpersonal neural synchronization (INS) between interlocutors in successful verbal communications. The opportunities for communication across different language contexts are rapidly expanding, thanks to the frequent interactions among people all over the world. However, whether the INS changes in different language contexts and how language choice affects the INS remain scarcely explored. The study recruited twenty pairs of participants to communicate in the first language (L1), second language (L2) and interlingual contexts. Using functional near-infrared spectroscopy (fNIRS), we examined the neural activities of interlocutors and analyzed their wavelet transform coherence to assess the INS of dyads. Results showed that as compared to the resting state, stronger INS was observed at the left inferior temporal gyrus, middle temporal gyrus, pre-motor and supplementary motor cortex, dorsolateral prefrontal cortex, and inferior frontal gyrus in L1; at the left middle temporal gyrus, superior temporal gyrus, and inferior frontal gyrus in L2; at the left inferior temporal gyrus and inferior frontal gyrus in interlingual context. Additionally, INS at the left inferior frontal gyrus was significantly stronger in L2 than in L1. These findings reveal the differences of the INS in different language contexts and confirm the importance of language choice for the INS changes.

## Introduction

Verbal communication is fundamental in human social interaction^1, 2^. Currently, researchers have begun to explore verbal communication with the synchrony of brain activity in temporal and spatial patterns, which is termed between-brain neural coupling^3^, interbrain coupling^4^, or interpersonal neural synchronization (INS)^5^. An accumulating number of studies have highlighted the importance of INS between interlocutors in successful verbal communications^1, 2, 6, 7^.

As alignment theories for interpersonal verbal communication stated^2, 7, 8^, communication is successful to the extent that interlocutors synchronize at different levels. The INS is considered to reflect mutual understanding between the listener and speaker, and represents a crucial neural mechanism for successful verbal communication^2, 7^. Previous studies have found that brain activities were closely coupled between the speaker and listener when they successfully communicated^3, 4, 6, 9^. These studies demonstrated that significant INS occurred both in areas associated with linguistic processes (e.g. the middle temporal gyrus, and the superior temporal gyrus) and in areas responsible for other social processes (e.g. the precuneus and the medial prefrontal cortex)^3, 9^.

However, whether the INS occurs or changes in different language contexts and the possible role of this change in communicative success in L2 and interlingual communications remain scarcely explored. In the globalized context, growing interactions between people from different parts of the world and rapid advancement of technologies have given rise to spoken communications in different language contexts. Communications in a second language (L2) or interlingual communications assisted by interpretation draws great attention^10, 11^. Thus, it is necessary to study the INS in various language contexts, namely, first language (L1), L2 and interlingual context.

Up to date, the investigation of INS in communicative settings has been mainly restricted to L1 context^1^. Compared with L1, L2 and interlingual contexts represent circumstances where intelligibility is blocked and the cognitive cost of producing and comprehending the language is higher^12^. For one thing, due to the fact that language processing involves different layers and various language contexts recruit different language systems, L2 and interlingual contexts will exhibit some different INS patterns that are associated with distinct linguistic levels for understanding and language specialty. For another, more cognitive resources are consumed for L2 and interlingual contexts if communicators tend to achieve similar comprehension level as to that of L1^13^. Thus, the INS patterns will also demonstrate some divergences relevant to cognitive resources such as working memory in different language contexts.

The hierarchical view of language was dated back to grammarians in the thirteenth century who analyzed a sentence into a subject and a predicate^14^. Later, researchers studied language-related neural activities at various linguistic levels, ranging from the lower levels of phonetics and phonemes^15^, to higher levels of words, sentences and texts^16–18^. Although there is no consensus on a general neural language network, hierarchical linguistic structures seemed to have been accepted and account for a multitude of research statistics^19^. For example, converging evidence has shown that speech is processed along the auditory pathways hierarchically^20, 21^. The dorsal stream of the superior temporal gyrus (STG) areas involves in initial acoustic analysis while the ventral stream of the superior temporal sulcus (STS) or middle temporal gyrus (MTG) regions is recruited for phonological processing^22^. Some researchers suggested a more macro hierarchical structure for language network, such as the 3-layered structure of pragmatics, semantics and phonology^23^. Language processing in L2 and interlingual contexts differs from that in L1 for interlocutors. As such, we hypothesized that the hierarchical nature is reflected in the INS in different language contexts.

Different languages differ in many ways including phonology, orthography, morphology, and syntax. Previous studies have implicated that the processing of different languages is associated with distinct cortical areas^24, 25^. The divergences of neural activities between languages involve phonological processing^24^, semantic processing^26^, syntactic processing^27^, and pitch processing^28^. Furthermore, the differences were found not only between the alphabetic language (such as English) and the logograph language (such as Chinese)^24^, but also between Indo-European languages such as French and Spanish^29^. Thus, we hypothesized that language specificity contribute to the different neural synchronization patterns in communications that occur in different languages.

Working memory (WM) processes and stores information online and is consequently important for daily communication^30^. It is a resource closely linked with speech production as well as speech comprehension^31, 32^. Interlocuters could not process sentences without the presence of working memory. However, L2 interlocutors do not have identical lexical and grammatical knowledge of the target language. They have difficulties in processing the L2 information, especially when the task is done online with time pressure^33^. In L2 communication, the lower-level language processing such as lexical access demand more cognitive resources, WM included, than that of L1 communication. Consequently, fewer resources are spared for higher-level constructions. Considering all, we hypothesized that foreign language deployment will interact with WM and differentiate the INS pattern in distinct language contexts.

Left hemisphere is traditionally regarded critical for language processing. Previous two-brain studies in L1 verbal communication have identified a variety of left cortical areas that contribute to INS, including the premotor cortex, supplementary motor cortex^9^, middle temporal gyrus^9, 34^, superior temporal gyrus^3, 9, 34, 35^, temporoparietal junction^3, 9^, parietal lobule^3^, and angular gyrus^9, 36^. Studies of WM have also suggested the left frontal cortex and parietal cortex are essential for WM network^37, 38^.

To the end, we examined INS increase between interlocutors when they communicated in L1 (Chinese to Chinese, CC), L2 (English to English, EE), and across languages with the assistance of interpretation (Chinese to English, CE) in left frontal, temporal and parietal cortices. The turn-taking verbal exchange was partially like a radio conversation to avoid overlap between the speaker and listener while interacting with others orally^4^ and to reduce the influence of translation quality. In the study, functional Near Infrared Spectroscopy (fNIRS) was employed to measure changes in hemoglobin concentration in an ecologically valid context because of its portability and tolerance for movement artifacts when simultaneously recording activities of two or more brains^1, 39^. Besides, fNIRS is superior in spatial resolution (10–20 mm) as compared with other hyperscanning techniques, such as EEG^40^. A comparison of inter-brain synchronization between conditions and the resting state was conducted separately, and subsequently, a between-condition comparison of the INS was performed.

## Results

### Behavioral results

Overall accuracy in the judgment for the understanding test was high (mean: 0.884, SD: 0.090), indicating a high level of communication quality or mutual understanding between interlocutors. The two-way repeated-measures ANOVA showed no significant effects of participant or communication condition, neither was there a significant interaction between participant and communication condition for accuracy (*P*s > 0.05). These results indicated that both Participants A and C communicated successfully in all the tasks, and similar successful understanding was achieved regardless of the language contexts.

**Figure 1:**
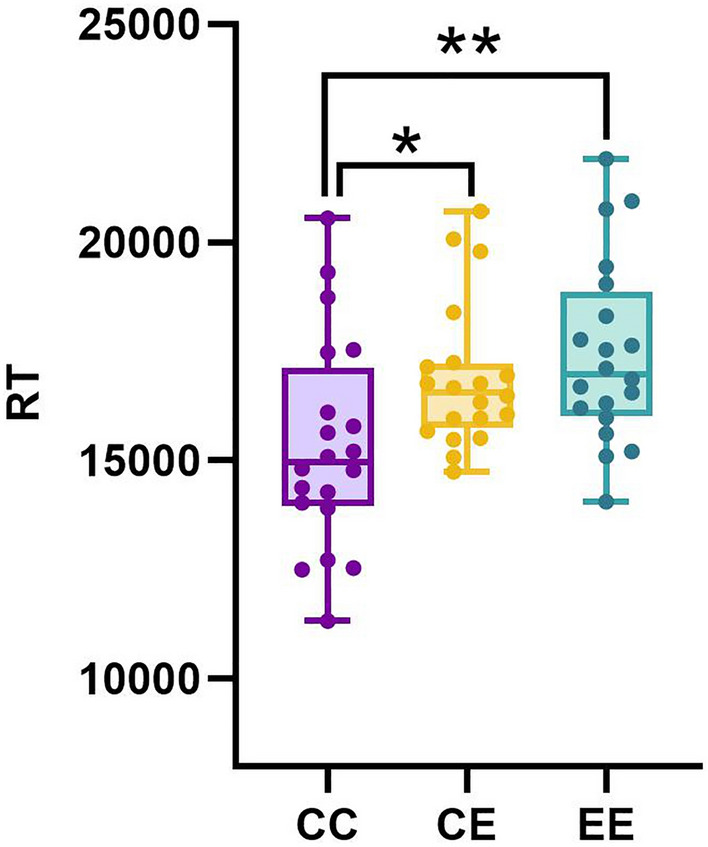
Comparisons of RT between L1, L2 and interlingual contexts when making the true-or-false judgements. Shorter RT for L1 condition than for interlingual condition, shorter RT for L1 condition than for L2 condition. *RT* reaction time, *CC* L1 context, *CE* interlingual context, *EE* L2 context. *p < 0.05, **p < 0.01.

The two-way repeated-measures ANOVA for reaction time (RT) showed that there was a significant participant and communication interaction (*F*(2, 76) = 3.267, *p* = 0.044). Further simple effect analysis showed that in CE condition, the RT of Participant A in L1 (15,830.550 ± 3501.494 ms) was significantly shorter than that of Participant C using L2 (17,957.526 ± 2398.684 ms) (*p* = 0.031). There was a significant main effect of communication condition (*F*(2, 78) = 6.616, *p* = 0.002). Pairwise comparisons (Fig. 1) showed that participants made the true-or-false judgments with significantly shorter RT in CC condition than in EE condition (*p* = 0.003, M_CC_ = 15,340.088 ms, SD_CC_ = 3130.878; M_EE_ = 17,454.668 ms, SD_EE_ = 3160.224), and in CE condition (*p* = 0.029, M_CE_ = 16,894.038 ms, SD_CE_ = 3152.163). Null significance was found for the RT between EE and CE conditions (*p* = 1.000). There was no main effect of participant (*F*(1, 38) = 0.434, *p* = 0.514). These findings indicated the conversations were successful in information exchange but communication with L2 (English) involved needed more time for processing than that with L1.

### fNIRs results

We conducted a paired *t*-test between the z-scored INS value of the individual task condition and the resting-state session. Later, a one-way repeated-measures ANOVA was performed on the z-scored INS value of the three conditions.

#### INS increase in L1, L2 and interlingual contexts as compared to the resting state

**Figure 2:**
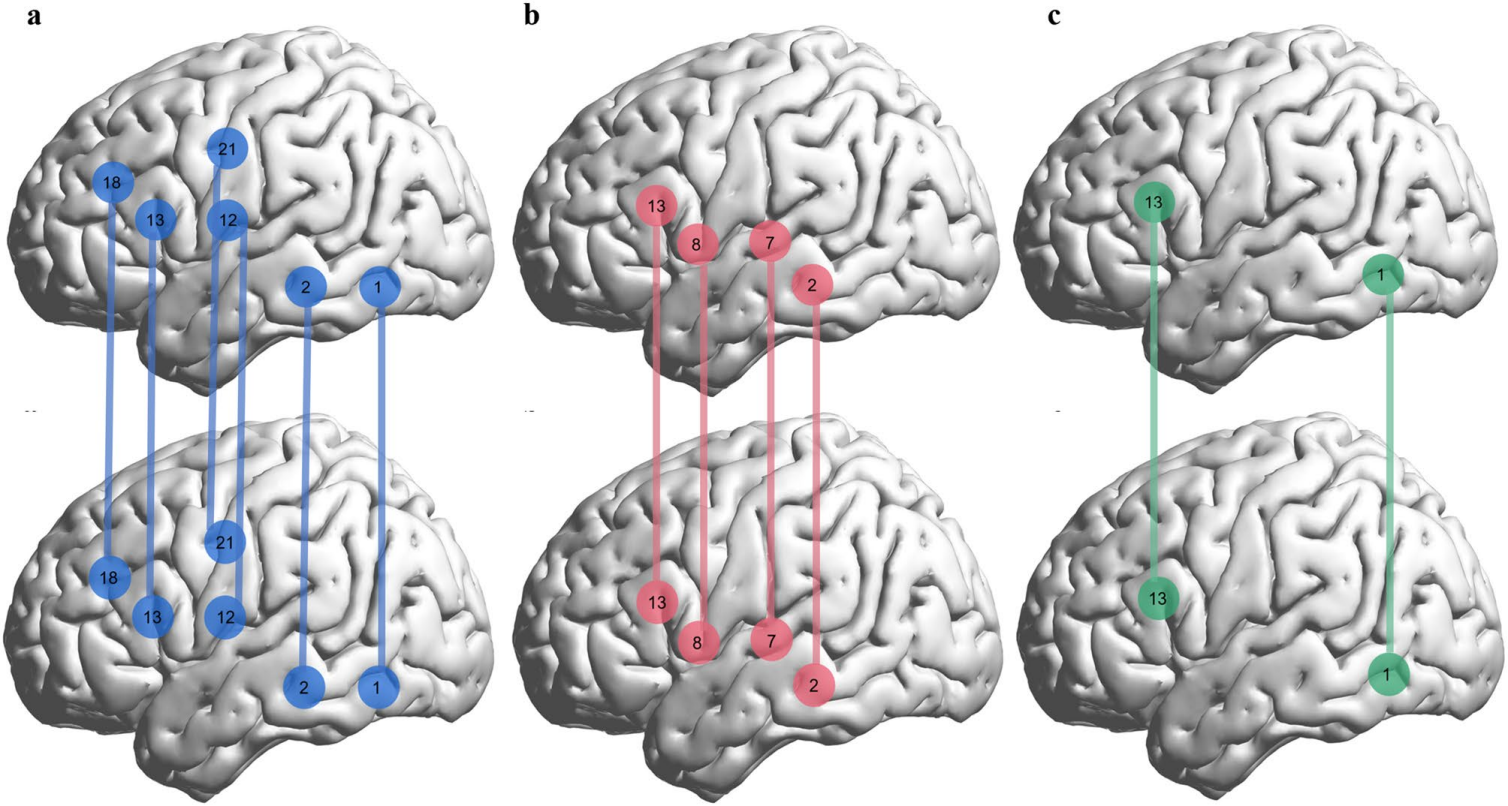
Channels where INS increase was significant in three conditions compared with the resting state. (**a**) INS increased for L1 condition in Channels 1, 2, 12, 13, 18, 21 in the left hemisphere. (**b**) INS increased for L2 condition in Channels 2, 7, 8, 13 in the left hemisphere. (**c**) INS increased for interlingual condition in Channel 1, 13 in the left hemisphere. (Figure was created with a Matlab toolbox, BrainNet Viewer Version 1.6.1, https://www.nitrc.org/projects/bnv/).

Synchronization between the speaker and the listener under three conditions was compared with the resting state separately. A significant greater INS increase was observed in interlingual condition, English (L2), as well as Chinese (L1). However, the cortical regions where larger INS emerged exhibited differences (Fig. 2) compared to those areas observed in L1. Participants showed stronger INS when interlocutors communicated in L1 at the inferior temporal gyrus (ITG, BA20, CH1, *t* = 2.967, *p* = 0.038), the middle temporal gyrus (MTG, BA21, CH2, *t* = 2.883, *p* = 0.038), the pre-motor and supplementary motor cortex (SMA, BA6, CH12, *t* = 2.851, *p* = 0.038; CH21, *t* = 3.168, *p* = 0.037), the inferior frontal gyrus (IFG, BA45, CH13, *t* = 3.845, *p* = 0.014), and the dorsolateral prefrontal cortex (dlPFC, BA46, CH18, *t* = 2.739, *p* = 0.042) in the left hemisphere (Fig. 2a). When they communicated in English (L2), the strong coupling between interlocutors was at the middle temporal gyrus (MTG, BA21, CH2, *t* = 3.503, *p* = 0.021), the superior temporal gyrus (STG, BA22, CH7, *t* = 3.712, *p* = 0.021), and the inferior frontal gyrus (IFG, BA45, CH8, *t* = 3.275, *p* = 0.025; CH13, *t* = 3.046, *p* = 0.035) in the left hemisphere (Fig. 2b). Finally, in interlingual communication, the large coupling was only found at the interior temporal gyrus (ITG, BA20, CH1, *t* = 3.114, *p* = 0.050) and the inferior frontal gyrus (BA45, CH13, *t* = 3.276, *p* = 0.050) (Fig. 2c).

#### INS increase in L2 as compared to L1

**Figure 3:**
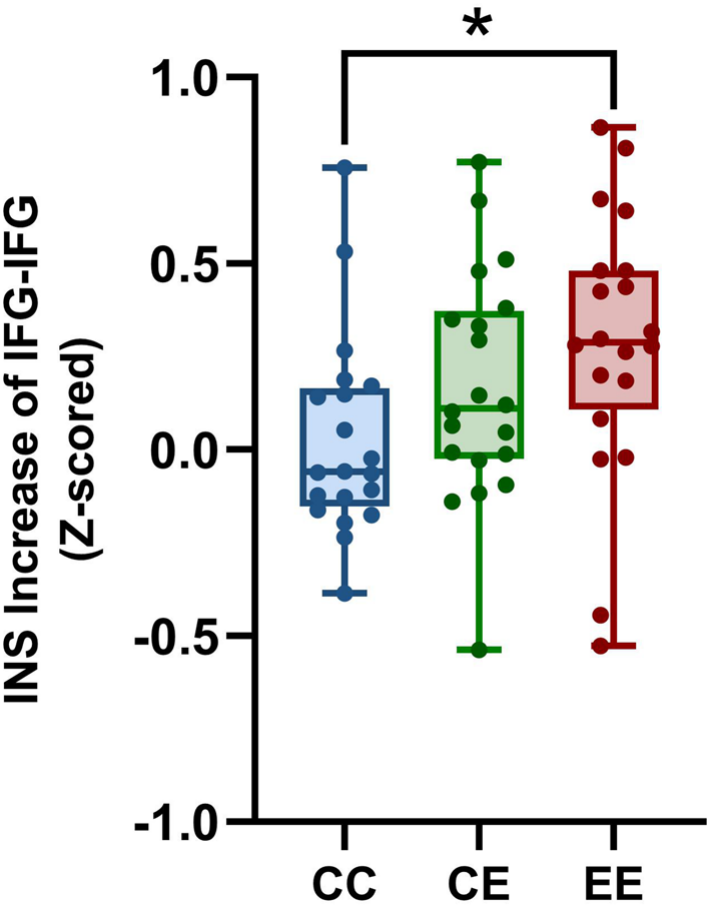
Comparisons of INS increase between L1, L2 and interlingual contexts. Stronger INS increase at the left inferior frontal gyrus (IFG) in L2 than in L1. *CC* L1 context, *CE* interlingual context, *EE* L2 context. *p < 0.05.

The one-way repeated-measures ANOVA on HbO across the communication conditions showed a significant effect on CH13 (*F*(2,38) = 8.928, *p* = 0.017, false discovery rate (FDR) corrected). Further pairwise analysis showed that the INS increase was significantly higher in L2 condition compared to L1 condition (*p* = 0.012) at CH13 (left IFG) (Fig. 3). No significant difference was observed between L1 condition and interlingual condition or between L2 condition and interlingual condition at CH13, nor were there any other significant differences at any other CHs.

The data of HbR concentration were also analyzed. No significant effect of communication condition was observed for HbR concentration.

## Discussion

The study examined the neural synchronization between the speaker and the listener in different language contexts. The results revealed that the INS channels varied for three conditions as compared to the resting state, suggesting the hierarchical nature of language processing and language specificity affect interlocutors’ similarity of the neural patterns in verbal communications. Moreover, the INS increase was significantly stronger at the left IFG during L2 interaction than during L1 interaction, where verbal working memory might function. These findings are discussed below.

First, the cortical areas with an increase in INS showed dissimilarities for three conditions. One possible account for the discrepancies might be the hierarchical nature of language processing. Language processing involves hierarchical layers, such as, phonology, semantics and pragmatics^23^. INS increase in L1, L2 and interlingual interactions showed distinct speaker-listener engagement for different layers.

At the phonological level, different from other conditions, communications in L2 particularly showed the INS increase at the left STG. The neural coupling between speech production and comprehension in English in this region was also observed in previous research^3, 9, 34, 35^. Their studies adopted a one-way communication paradigm, investigating “information flow” from the brain of the sender (the speaker) to the brain of the receiver (the listener). Otherwise, ours developed it further to two-way communication, in which the interlocutors exchanged their roles of both the speaker and listener. The INS might be due to the involvement of the STG in the external loop of self-monitoring as researchers suggested^3, 9^. Some additional accounts might also contribute to the finding. According to Hickok and Poeppel^41^, in their dual-stream model of speech, the STG is proposed to be involved in spectrotemporal analysis. Moreover, it was proved that the STG is associated with lexical phonological encoding^42^. The STG also played an important role for phonology processing in speech production. Thus, it is not surprising to find INS increase at the left STG in L2 (English) communication, implicated as interlocutors’ brain-to-brain synchronization at the phonological level.

At the semantic level, we found a significant INS increase at the left MTG in L1 and L2 conditions against the resting state, at the left ITG in L1 and interlingual conditions. The findings were accounted for as production-comprehension alignment at semantic level. The left MTG serves for lexical access^43^ and sentence processing^44^. These are necessary for both production and comprehension. The left MTG was also found interconnected with many language-related cortical regions in frontal, parietal and temporal lobes with white matter pathways^45^. The INS exhibited in this region was similarly found in previous study when participants produced and comprehended naturalistic narrative speech^9^. Taken together, the INS increase observed at the left MTG implicated dyads understood each other well in both L1 and L2.

The ITG is involved in semantic processing as well. Sharp et al.^46^ have observed greater activation in the ITG when processing the meaning of single words than rotated speech baseline. It has also been suggested to be responsible for semantic storage and grammatically correct sentence discrimination^47^. In addition, meta-analyses suggested the ITG is involved in both language reception and understanding in lexical-semantic system^48^. Accordingly, the robust INS increase during L1 and interlingual conversations in the present study might signify that the dyads achieve mutual understanding at semantic level, sharing content irrespective of language forms.

At pragmatic level, only participants in L1 presented the INS increase at higher-order, extralinguistic areas such as SMA and dlPFC. Previous research indicated that the SMA was involved in emotion^49^ and empathy^50^, which might be required for pragmatic context integration with semantic tasks. While, as to dlPFC, previous studies claimed it a key region for pragmatic processing. The dlPFC was activated in a range of tasks where pragmatic involvement is necessary, such as sarcastic sentences^51^ and metaphors^52^. Moreover, the dlPFC was engaged in discourse coherence^53^. Our findings of INS increase at dlPFC were consistent with that in previous one-way communication of stories^3^. Pérez et al.^4^ also discovered stronger INS in the alpha band in fronto-central areas in L1 than in L2. The coupling pattern in these areas between the speaker and listener in L1 might suggest higher-level processing of complex dynamic information exchange between two communicators.

To summarize, the widespread INS between interlocutors during L1 communication, covered the left SMA, dlPFC, MTG, and ITG. They were recruited for mutual understanding at linguistic levels of pragmatics and semantics. While INS was achieved in L2 at the left MTG and STG, showing similar inter-brain synchronization at semantic and phonological levels. Finally, in interlingual condition, the brain-to-brain coupling was found at the left ITG, indicating mutual understanding at semantic level. This trend might be yielded by different language codes in the experiment, Chinese as the first language, English as the second language. As previous research suggested, foreign language contexts represent a circumstance where intelligibility is blocked^4, 12^. Thus, although the behavioral statistics showed similar communication quality, the linguistic levels at which the listener synchronized with the speaker differed when language contexts changed.

Second, the INS in different language contexts exhibited language specificity to some extent. We found significant INS increase at the dlPFC in Chinse (L1) condition compared with the resting state, but no similar neural patterns in these regions in English (L2) condition as compared to the resting state. The dlPFC is overlapped with middle frontal gyrus, which was reported to have a larger involvement in Chinese character recognition than in English word recognition^24^. Other contrasts between Chinse and English provided compelling evidence similarly. For example, comparisons have found this region more involved in Chinese reading than English reading^54^. It also exhibits greater activation during handwriting for Chinese than for alphabetic^55^.

In contrast, significant INS increase was observed at the left STG in English against the resting state, but null in Chinese. Meta-analysis has found that the left STG is engaged in alphabetic languages more than in logographic ones^24, 54^. It was identified as a region critical for alphabetic word reading.

As evidenced above, the disparate regions for significant INS in Chinese and English might be attributed to language specificity of the involved ones. Our findings provided tentative evidence for language specificity with hyperscanning statistics.

Third, compared with L1 communications, L2 communications elicited significantly stronger INS increase at the left IFG. The IFG was previously discovered to correlate with working memory^37^. WM helps information to be held for much longer spans during which the subject rehearses the phonological information subvocally and repeatedly to avoid losing it^56^. Assisted by WM, communicators understood each other and pushed ahead with the communication under three conditions.

Abundant research has rectified the links of WM to language production and comprehension in L1^31, 32^ as well as in L2^57, 58^. However, WM was reported to show discrepancies in L1 and L2^59^, and there was an interplay between WM and (foreign) language proficiency^60^. Less WM resources are needed when comprehension of sentences improves with practice. L1 communicators are experts in their L1, so their L1 processing is automatic, especially when surface form and text base are involved^13^. They process L1 quickly and efficiently, and consume a small amount of cognitive resources. On the contrary, L2 interlocutors have more difficulties in processing the linguistic information under time pressure while communicating, for their inefficient lexical and grammatical knowledge of the target language ^33^. This can be supported by the behavioral findings in the present study. The reaction time for true-or-false judgment in L2 is significantly longer than in L1, even though there was no significant difference in the accuracy. Furthermore, our findings at the IFG might be an indicator of INS at the extra-linguistic level, recruitment of WM resources, supporting the generative theoretical model of interpersonal verbal communication^2^. When L2 was involved, to achieve a similar understanding level, the interlocutors allocated more cognitive resources to WM and showed stronger synchronization between them than in L1.

## Conclusions

As interactions between people across the world continue to increase, verbal communication in various language contexts is becoming more frequent. The language chosen for communication, whether it be the speaker's L1, L2, or facilitated through interpretation in an interlingual exchange, plays a crucial role in the verbal communication. In this study, we investigate how language choice affects the increase in INS during communication. The results indicated a more widespread network of INS during L1 communication compared to L2 and interlingual communication. Additionally, a greater INS was observed in the left inferior frontal gyrus (IFG) during L2 communication compared to L1. These distinct INS patterns can be attributed to different linguistic processes and the varied recruitment of working memory resources in different language contexts. By exploring INS in various language contexts, we can gain insights into neural activities and mechanisms that underlie successful interpersonal communication. This approach may shed light on the factors contributing to interactive verbal communication.

## Methods

### Participants

Forty healthy volunteers participated in the study (20 pairs, same gender in each dyad; 10 males; mean age = 22.77 years, SD = 1.63 years) and received monetary compensation. All participants are right-handed college students with normal or correct to normal vision. No hearing, neurological/psychiatric disorders or drug use was reported by the subjects. We choose to have same-sex participants in each dyad, rather than mixed-sex interlocutors, for sex might moderate human social interactions and brain activations^61^. To minimize the impact of L2 proficiency on communicative success, only participants who scored higher than 75 on the TEM4 were recruited. The TEM4 is a national test in China that assesses the English proficiency of college students majoring in English and determines whether they meet the required levels of listening, reading, and writing as described in the National College English Teaching Syllabus for English Majors^62^. Based on statistics from test takers at a university in our city in 2021, a score of 75 represents good proficiency, as it falls at the 25th percentile. Those with strong accents were excluded after reading a paragraph in English. Written informed consent was obtained from all subjects. The study protocol was approved by the Ethics Committee on Human Experimentation for Key Laboratory for Artificial Intelligence and Cognitive Neuroscience of Language, Xi’an International Studies University (protocol code 2021-AICNL-f002 and date of approval: 18 July 2021).

### Experimental procedure

**Figure 4:**
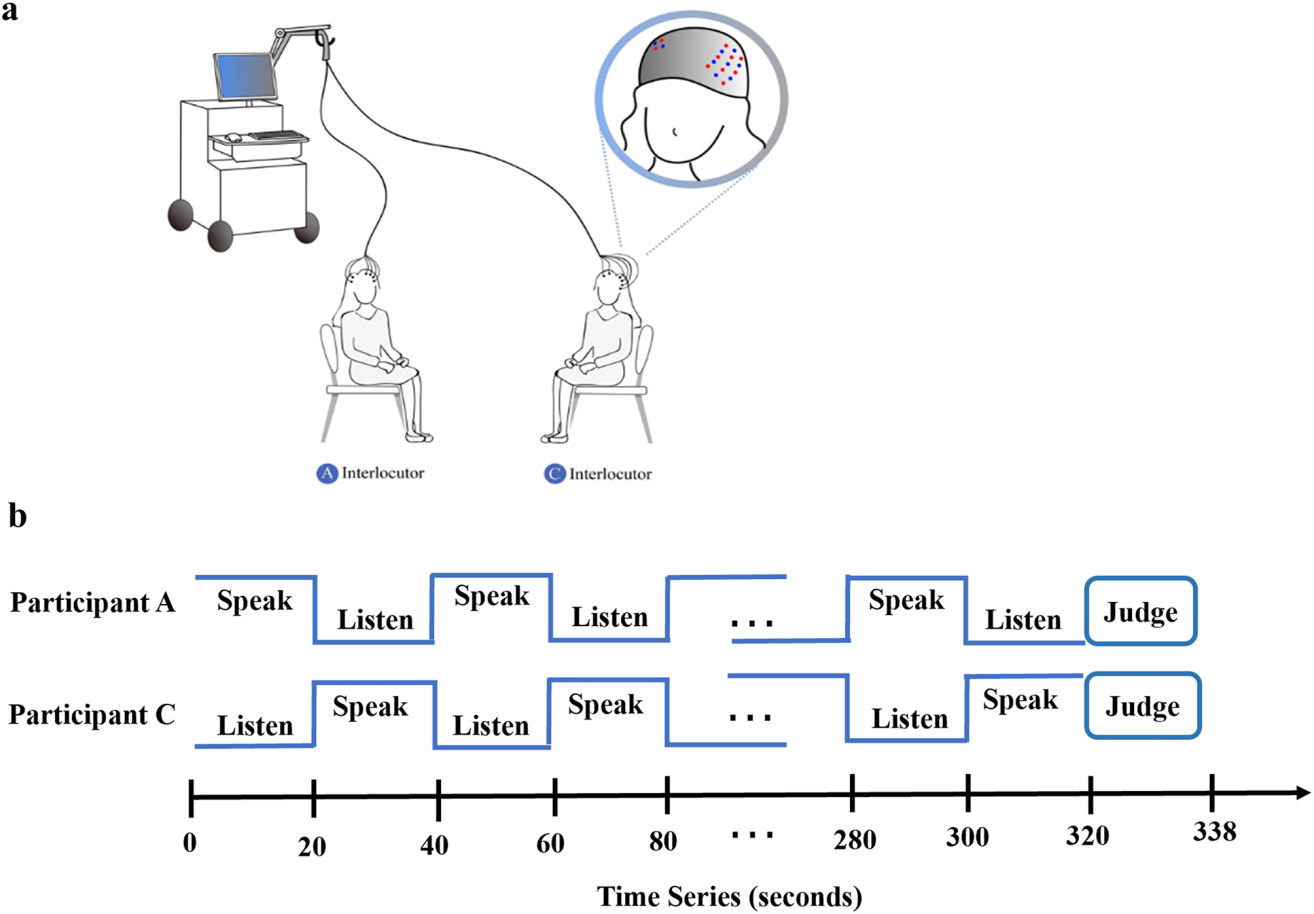
fNIRS setup and experimental paradigm. (**a**) Two participants sat with a screen in front of each to signify the procedure and to present material for utterance and questions for judgment. (**b**) The experimental procedure for one topic. (Figure (**a**) was created with Adobe Illustrator 2020, https://www.adobe.com/products/illustrator/free-trial-download.html).

The experiment was conducted in a quiet room (Fig. 4a). Participants were initially required to complete a 5-min resting-state session as a baseline, during which they kept their minds relaxed with eyes closed, and tried to minimize movements^5^. After the resting-state session proceeded the communication session.

In total, each pair was required to conduct turn-taking verbal communications under three conditions. Under condition 1, both dyadic partners (Participants A and C) communicated verbally in their native Chinese. Under condition 2, the procedure was the same except that both interlocutors exchanged information in English, L2 to them. Under condition 3, one communicator (Participant A) adopted Chinese while his partner (Participant C) utilized English, in an interlingual context. To counterbalance the sequence of three communication conditions across participant pairs, seven dyads finished tasks in the order of CC, EE, CE; the other 7 in EE, CC, CE; and the rest 6 in CE, EE, CC. After each condition, participants had a rest until they felt it was efficient for recovery and the next task.

For each condition (L1, L2, and interlingual), two different common topics were presented, totaling six topics across all conditions. The topics included traveling and movies for the L1 condition, weekend plans and online courses for the L2 condition, and picnics and pets for the interlingual condition. Before each condition, one short example was provided to ensure participants’ familiarity with the procedure of the task. After each topic, three true-or-false questions were presented on the screen for communicators to judge, which was designed to determine whether the subjects involved understood the communication well and whether different contexts ignited different difficulty levels for participants. Participants had to finish each judgement with 6 s, otherwise, it would be regarded as a wrong answer. In all conditions, participants in each dyad were required to alternate between the “speaker” and “listener” roles every 20 s (Fig. 4b). This exchange between talking and listening continued for 320 s for each topic.

Conversations began with Participant A and a cue of “ding” sound indicated which member of the dyad had to speak or to listen. When the “ding” sound rang, Participant A saw sentences appearing on the screen with a 20-s countdown, and they were required to verbalize the content on the screen. Meanwhile, Participant C saw a speaker icon on the screen and was required to listen carefully via microphones. Here we take a short practice sentence for instance. When Participant A said “Today is my sister's graduation day, and I bought some flowers for her.” as cued by the screen, Participant C would hear the sentence's recording through a microphone. Then, Participant C would see “It must have cost you a fortune. It is normally very expensive.” on their screen and speak within 20 s, while Participant A heard its recording through a microphone. In the L1 condition, all materials were in Chinese, either visually or auditorily, while in the L2 condition, all materials were in English. In the interlingual condition, Participant A saw, spoke, and heard everything in Chinese, while Participant C saw, spoke, and heard everything in English.

This procedure ensured that Participant A heard (or spoke) the same content in Chinese exactly when Participant C spoke (or heard) it in English in an interlingual context without any time lags between the communicating partners’ articulatory and auditory processes, as temporal process profoundly impacts the INS research^7, 34^. Additionally, adopting the interpretation recorded in advance eliminated the influence of interpretation quality, avoiding a direct hearing of the dyadic participant in an interlingual context, which can significantly impact their neural activities.

### fNIRS data acquisition

**Figure 5:**
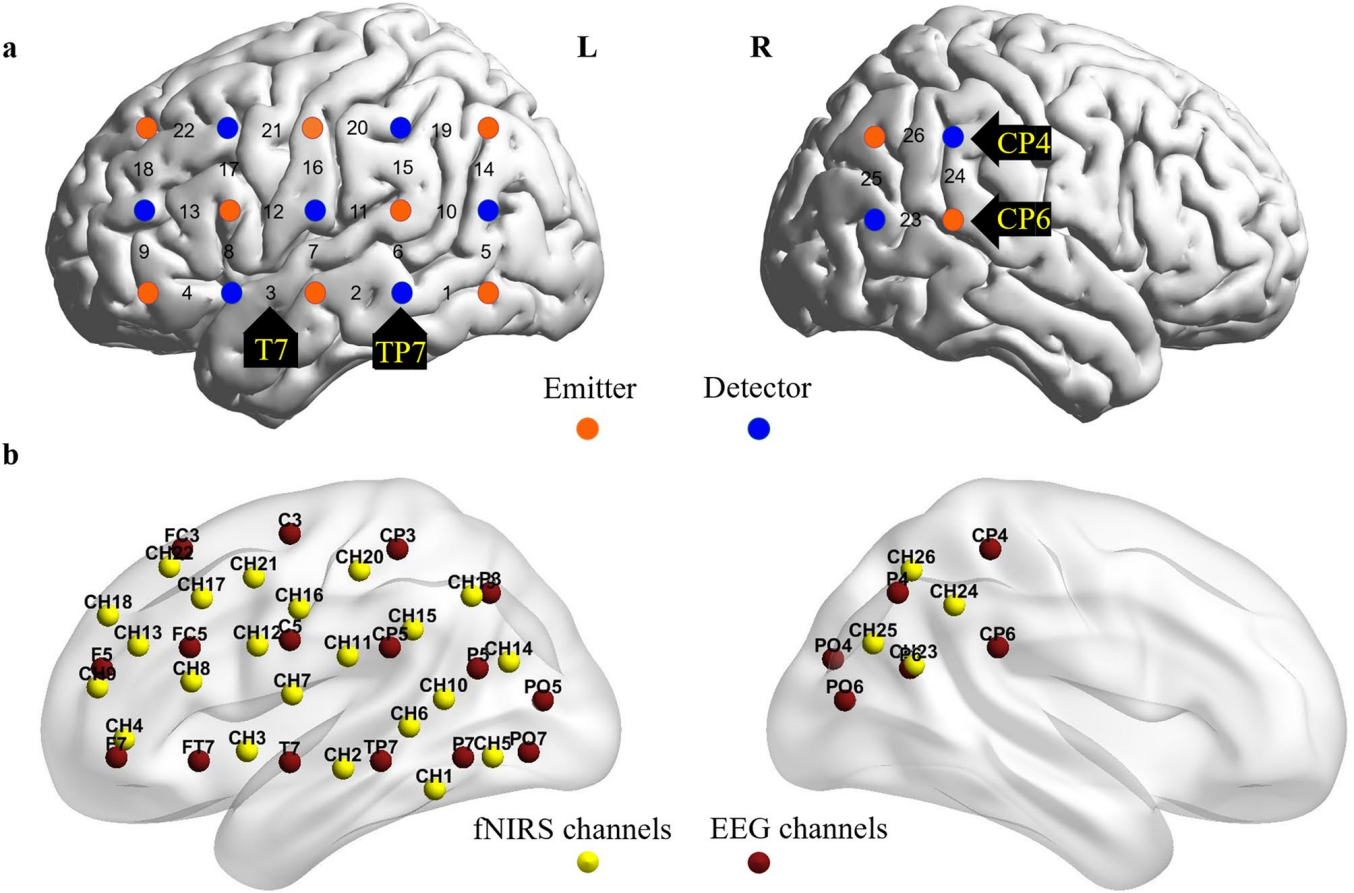
Channels in the left and right hemispheres. (**a**) On the left hemisphere was a 3 × 5 optode probe set (8 emitters and 7 detectors, 22 channels). CH3 and the bottom right detector were placed at T7 and Tp7, respectively, following the international 10–10 system. On the right hemisphere was a 2 × 2 optobe probe set ((2 emitters and 2 detectors, 4 channels). The top right and bottom right optodes were placed at Cp4 and Cp6, respectively, in accordance with the international 10–10 system. (**b**) Correspondence between each channel and the international 10–10 system positions. *L* left hemisphere, *R* right hemisphere. (Figure was created with a Matlab toolbox, BrainNet Viewer Version 1.6.1, https://www.nitrc.org/projects/bnv/).

A continuous wave fNIRS system (ETG-7100 Hitachi Medical Tokyo, Japan) was employed to measure cortical oxyhemoglobin, deoxyhemoglobin, and hemoglobin signals. Each participant had two optode probe sets placed on them. One probe set consisted of a 3 × 5 array of optodes with 8 emitters and 7 detectors, resulting in 22 channels. This set was positioned on the left frontal, temporal, and parietal cortices, with the middle optode of the lowest probe row and the bottom right detector placed at T7 and Tp7 respectively in accordance with the international 10–10 system. Another probe set, consisting of 2 emitters and 2 detectors with 4 channels, was used to cover the right temporal-parietal (rTPJ) region (Fig. 5a). However, this set was for other research questions and was reported in a separate paper. The top right and bottom right optodes were placed at Cp4 and Cp6, respectively, in accordance with the international 10–10 system. The distance between each emitter and detector was 3 cm. The correspondence between each channel and the international 10–10 system positions was showed in Fig. 5b.

A 3-D magnetic space digitizer device (FASTRAK; Polhemus, United States) was used to capture the 3-D spatial coordinates of each optode placed on the participant’s scalp. Each channel’s corresponding coordinates in the Montreal Neurological Institute (MNI) was estimated using a probabilistic registration method from NIRS_SPM software. The mean 3D MNI coordinates and associated brain regions of the 26 channels were provided in Table 1.

**Table 1:** The mean 3D MNI coordinates and associated brain regions of the 26 channels.

Channels	Hemisphere	x	y	z	Brodmann area	Probability
CH01	Left	− 67	− 48	− 15	20—Inferior Temporal gyrus	0.600
CH02	Left	− 71	− 21	− 9	21—Middle Temporal gyrus	0.947
CH03	Left	− 61	6	− 4	48—Retrosubicular area	0.410
CH04	Left	− 53	42	0	45—pars triangularis Broca's area	0.613
CH05	Left	− 62	− 64	− 5	37—Fusiform gyrus	1.000
CH06	Left	− 70	− 40	3	22—Superior Temporal Gyrus	0.533
CH07	Left	− 67	− 7	13	22—Superior Temporal Gyrus	0.441
CH08	Left	− 59	22	16	45—pars triangularis Broca's area	0.513
CH09	Left	− 47	49	15	46—Dorsolateral prefrontal cortex	0.607
CH10	Left	− 68	− 50	11	22—Superior Temporal Gyrus	0.476
CH11	Left	− 69	− 23	24	2—Primary Somatosensory Cortex	0.495
CH12	Left	− 64	3	27	6—Pre-Motor and Supplementary Motor Cortex	0.503
CH13	Left	− 49	38	27	45—pars triangularis Broca's area	0.963
CH14	Left	− 58	− 69	22	39—Angular gyrus, part of Wernicke's area	0.769
CH15	Left	− 67	− 41	31	40—Supramarginal gyrus part of Wernicke's area	0.535
CH16	Left	− 64	− 9	37	43—Subcentral area	0.460
CH17	Left	− 52	19	40	44—pars opercularis, part of Broca's area	0.741
CH18	Left	− 37	46	35	46—Dorsolateral prefrontal cortex	0.676
CH19	Left	− 59	− 58	41	39—Angular gyrus, part of Wernicke's area	0.675
CH20	Left	− 62	− 26	48	3—Primary Somatosensory Cortex	0.292
CH21	Left	− 54	4	46	6—Pre-Motor and Supplementary Motor Cortex	0.870
CH22	Left	− 39	29	49	9—Dorsolateral prefrontal cortex	0.930
CH23	Right	64	− 59	21	22—Superior Temporal Gyrus	0.443
CH24	Right	65	− 47	38	40—Supramarginal gyrus part of Wernicke's area	0.882
CH25	Right	56	− 71	27	39—Angular gyrus, part of Wernicke's area	1.000
CH26	Right	56	− 60	48	39—Angular gyrus, part of Wernicke's area	0.657

Data from individual channels were collected at two different wavelengths, 695 nm and 830 nm at a sampling rate of 10 Hz. The changes in the oxyhemoglobin (HbO) and deoxyhemoglobin (HbR) were calculated based on the modified Beer-Lambert Law. In this study, HbO concentration changes were focused in the following statistical analysis because HbO was previously validated as the most sensitive signal to hemoglobin changes^5, 39^.

### Behavioral data analysis

To investigate the quality of communication across interlocutors and communication conditions, we conducted a two-way repeated-measures ANOVA with a 2 × 3 design. Accuracy and reaction time for judging true-or-false questions were the dependent variables. The between-subjects factor was participant (Participant A and Participant C), while the within-subjects factor was communication condition (CC, EE, and CE).

### fNIRS data analysis

Data collected during the resting state and task session were analyzed by using Wavelet Transform Coherence (WTC) to identify the cross-correlation between two fNIRS time series generated by a pair of participants as a function of both frequency and time^39, 63^. The HbO time series of each channel from each pair of participants were obtained simultaneously, then WTC was conducted to the two time series, generating a 2D matrix of the coherence values. Its column and row represent specific frequencies and time points. All the coherence values were converted into Fisher z-values and averaged across time series. In order to remove the high- and low-frequency noises, such as those associated with respiration (about 0.2–0.3 Hz) and cardiac pulsation (about 1 Hz), a frequency period of 10–40 s (corresponding to frequency 0.025–0.1 Hz, respectively) was selected for statistical analyses.

To test that the validation of INS increase was specific to the linguistic context, permutation test was applied. Participants were randomly grouped to form new pairs, and INS increase was recalculated. Paired *t*-tests were applied to the INS increase. The permutation test was carried out 1000 times to produce a distribution (F value) of all CHs, which was then compared with the original data. A false discovery rate (FDR) method (*p* < 0.05) was used for correction.

A paired *t*-test was finally performed between the individual task and resting-state session separately. A one-way repeated-measures ANOVA was conducted on the INS increase over all CHs, in which the within-subjects factor is communication condition (CC vs. EE vs. CE).

### Ethical approval

The study protocol was approved by the Ethics Committee on Human Experimentation for Key Laboratory for Artificial Intelligence and Cognitive Neuroscience of Language, Xi’an International Studies University (protocol code 2021-AICNL-f002 and date of approval: 18 July 2021). The study was performed in accordance with the Declaration of Helsinki. Written informed consent was obtained from all participants.

## Data Availability

The data generated or analyzed during this study in this study will be shared on reasonable request to the corresponding author.
